# Bioinformatic Analysis and In Vitro and In Vivo Experiments Reveal That Fibrillarin Participates in the Promotion of Lung Metastasis in Hepatocellular Carcinoma

**DOI:** 10.3390/bioengineering9080396

**Published:** 2022-08-17

**Authors:** Weixin Luo, Shusheng Lin, Yipei Huang, Ke Zhu, Fapeng Zhang, Junlong Lin, Yufei Qin, Ziyu Zhou, Wenrui Wu, Chao Liu

**Affiliations:** 1 Department of Biliary-Pancreatic Surgery, Sun Yat-sen Memorial Hospital, Sun Yat-sen University, Guangzhou 510120, China; 2 Guangdong Provincial Key Laboratory of Malignant Tumor Epigenetics and Gene Regulation, Sun Yat-sen Memorial Hospital, Sun Yat-sen University, Guangzhou 510120, China

**Keywords:** fibrillarin, hepatocellular carcinoma, lung metastasis, bioinformatic analysis, WGCNA

## Abstract

Lung metastasis, the most frequent metastatic pattern in hepatocellular carcinoma, is an important contributor to poor prognosis. However, the mechanisms responsible for lung metastasis in hepatocellular carcinoma remain unknown. Aiming to explore these mechanisms, weighted gene coexpression network analysis (WGCNA) was firstly used to find hub genes related to lung metastasis. Then, we obtained 67 genes related to lung metastasis in hepatocellular carcinoma which were mainly related to ribosomal pathways and functions, and a protein interaction network analysis identified that fibrillarin (FBL) might be an important hub gene. Furthermore, we found that FBL is highly expressed in hepatocellular carcinoma and that its high expression increases the rate of lung metastasis and indicates a poor prognosis. Knockdown of FBL could significantly reduce proliferation and stemness as well as inhibiting the migration and invasion of hepatocellular carcinoma cells. Moreover, we found that FBL might be involved in the regulation of MYC and E2F pathways in hepatocellular carcinoma. Finally, we demonstrated that the knockdown of FBL could suppress hepatocellular carcinoma cell growth in vivo. In conclusion, ribosome-biogenesis-related proteins, especially Fibrillarin, play important roles in lung metastasis from hepatocellular carcinoma.

## 1. Introduction

With the characteristics of an insidious onset, rapid progress, and poor prognosis, hepatocellular carcinoma is one of the leading causes of cancer-associated deaths worldwide [1]. Although more and more treatments for hepatocellular carcinoma are being developed, the 5-year overall survival rate of hepatocellular carcinoma patients has not improved significantly in the past 10 years, and one of the most important reasons for this is that hepatocellular carcinoma is very prone to metastasis [2,3,4]. The metastatic sites of hepatocellular carcinoma include the lungs, bones, adrenal glands, brain, lymph nodes, and peritoneum, among which lungs are the most frequent site [5,6]. However, the mechanism underlying lung metastasis from hepatocellular carcinoma is still not very clear.

Weighted gene coexpression network analysis (WGCNA) is a bioinformatics analysis method in which a coexpression network is constructed across the entire microarray or RNA sequencing data. It aims to find gene modules with coexpressed genes and to explore the relationship between gene modules and phenotypes of interest as well as the core genes in the network [7]. The Cancer Genome Atlas (TCGA) is one of the most widely used databases in cancer research today, and from this, many researchers have used the WGCNA method to discover a variety of molecular mechanisms involved in tumor development [8,9]. Although many studies have used hepatocellular carcinoma data from the TCGA to perform WGCNA analysis, no relevant research has investigated lung metastasis from hepatocellular carcinoma [10,11].

**Figure 1:**
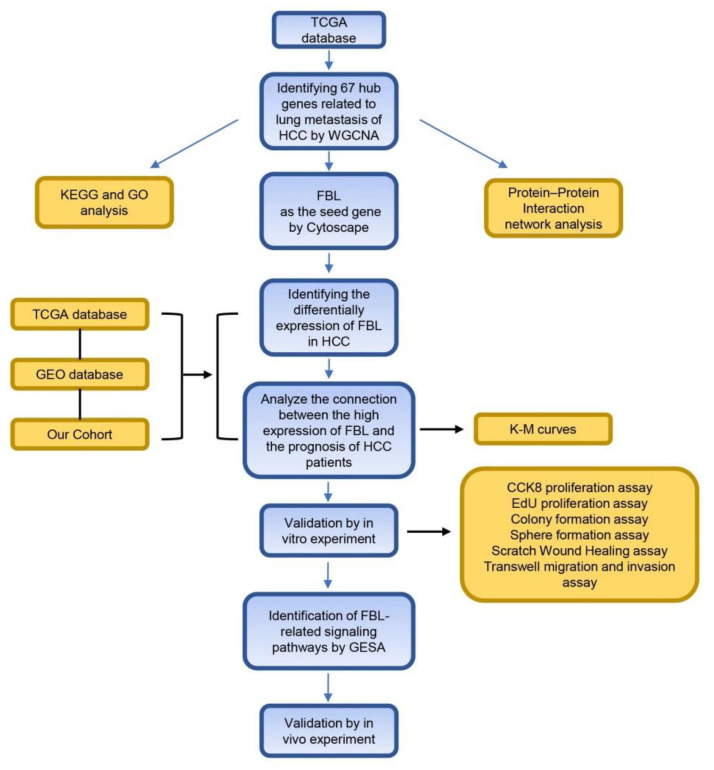
The flow diagram of this study.

In our study, WGCNA was firstly used to find the hub genes associated with hepatocellular carcinoma and lung metastasis by comparing hepatocellular carcinoma cases with and without lung metastasis in the TCGA. Then, we used the Gene Expression Omnibus (GEO) database and our own cohort to verify the impact of the FBL gene on the prognosis. In addition, in vitro or in vivo experiments were used to verify the effects of the FBL gene on the proliferation, stemness, migration, and invasion of hepatocellular carcinoma cells (Figure 1). Our findings may provide new ideas relevant to the exploration of the molecular mechanism responsible for lung metastasis in hepatocellular carcinoma.

## 2. Materials and Methods

### 2.1. Data Acquisition and Screening

We downloaded gene expression profile data and clinical data collected from patients with hepatocellular carcinoma from the TCGA database (https://portal.gdc.cancer.gov/projects/TCGA-LIHC, accessed on 27 February 2020). Transcriptome sequencing data of 370 hepatocellular carcinoma tissues and 47 normal liver tissues were obtained. We focused on the differences between samples of lung metastasis after hepatectomy and samples without recurrence or metastasis within 5 years after hepatectomy. According to the follow-up data, a total of 32 cases screened met the study’s requirements (including 18 cases of lung metastasis after hepatectomy and 14 cases of no recurrence or metastasis within 5 years after hepatectomy). We also downloaded the GSE14520 dataset and corresponding clinical information from the GEO database (https://www.ncbi.nlm.nih.gov/geo/, accessed on 4 April 2020), which contained 247 hepatocellular carcinoma samples and 241 normal liver tissue samples adjacent to cancer. Besides, hepatocellular carcinoma proteomes containing 165 samples were obtained from Proteomic Data Commons PDC000198 (https://pdc.cancer.gov/pdc/, accessed on 19 August 2020).

### 2.2. Construction of the Gene Coexpression Network

The WGCNA R package (version 1.69) was used to analyze the data [7]. Firstly, we obtained the top 5000 genes according to the median absolute deviation (MAD) size of each gene in the samples. Then, we clustered the samples, deleted outliers (TCGA.DD.A73G), and performed a gene coexpression network analysis on samples that met the criteria. A topological overlap matrix (TOM) was constructed with a suitable soft threshold β value (β = 9, scale-free R^2^ = 0.85). Genes were classified according to the hybrid dynamic shearing tree method, the minimum number of genes in each module was set at 30, and finally the differences of each module were calculated. A heatmap plot was used to visualize the topological overlap matrix (TOM) for all genes.

### 2.3. Identification of Significant Relevant Modules and Hub Genes

The gene modules related to lung metastasis were determined by the correlations between the modules and lung metastasis. In our study, modules whose *p* value was <0.01 were considered statistically significant. For statistically significant modules, we used the “signedKME” function in the WGCNA package (version 1.69) to calculate the module membership (MM) and gene significance (GS) in the module. The genes with MM > 0.8 and GS > 0.4 were screened as genes related to lung metastasis. Further, we used the pheatmap package (version 1.0.12) to display the differences in the expression of lung-metastasis-related genes in hepatocellular carcinoma tissues and normal liver tissues as well as the differences between hepatocellular carcinoma tissues with lung metastasis and those without metastasis.

### 2.4. Gene Ontology Annotations and Kyoto Encyclopedia of Genes and Genomes Pathway Analysis of the Hub Genes

The Clusterprofiler package (version 3.16.0) was used to annotate and enrich the signal pathway of lung-metastasis-related genes using the Kyoto Encyclopedia of Genes and Genomes (KEGG) database and the Gene Ontology (GO) database. This included biological processes (BPs), cellular components (CCs), and molecular functions (MFs). A *p* adjusted value of <0.05 was considered statistically significant [12].

### 2.5. Protein–Protein Interaction Network Analysis of the Hub Genes

The Search Tool for the Retrieval of Interacting Genes (STRING) database (version 11.0) (https://www.string-db.org, accessed on 16 April 2021) was used to analyze the interactions between the proteins of target genes to map the Protein–Protein Interaction network with a high level of confidence (minimum required interaction score = 0.700) [13]. The cytoHubba plug-in was used to screen the top 10 genes according to the Degree score, and the MCODE plug-in was used to screen the core subnetworks to determine the genes in the core area of the interaction network with the Cytoscape software.

### 2.6. Expression and Prognosis Analysis of the FBL in TCGA, GSE14520, and Our Cohort

We used the ggpubr package (version 0.4.0) in R to construct violin plots or box plots to show the difference between the expression of FBL in hepatocellular carcinoma tissues versus that in adjacent normal liver tissues. The Wilcoxon rank sum test was used for statistical testing, and *p* < 0.05 was judged to be statistically significant.

We used the survival package (version 3.1-12) and the survminer package (version 0.4.8) in R to calculate the cumulative survival time of patients through the Kaplan–Meier (KM) method, and the built-in Peto-Peto’s modified survival estimate method in the survminer package was used for statistical testing. The overall survival time was defined as the time from the start of surgery to death due to any cause or until the last follow-up. Disease-free survival was defined as the time from the start of the operation until recurrence of the disease or death (for any reason).

### 2.7. Clinical Specimens

All HCC tissues and normal liver tissues were collected from HCC patients who underwent liver resection at the Sun Yat-Sen Memorial Hospital, Sun Yat-Sen University. All procedures were conducted with the approval of the Ethical Committee of the Sun Yat-Sen Memorial Hospital, Sun Yat-Sen University. Patient consent was obtained before the collection of tissue samples.

### 2.8. Cell Lines and Culturing Conditions

The human hepatocellular carcinoma cell lines Huh7 and PLC/PRF/5, and the immortalized liver cell line LO2 were purchased from the America Type Culture Collection. The cell lines were cultured in Dulbecco’s modified Eagle’s medium (BI, Biological Industries Israel Beit Haemek Ltd., Beit Haemek, Israel) supplemented with 10% fetal bovine serum (GIBCO, Life Technologies Corporation, New York, NY, USA), 100 μg/mL penicillin, and 100 μg/mL streptomycin at 37 °C in a humidified incubator containing 5% CO_2_.

### 2.9. Western Blotting

Cells and tissue samples were harvested in RIPA lysis buffer containing protease and phosphatase inhibitors to obtain protein extracts. A bicinchoninic acid (BCA) assay was performed to measure the protein concentrations. Then, equal amounts of the extracts were separated in an SDS-polyacrylamide gel, electrophoretically transferred to the polyvinylidene fluoride membrane (Millipore, Merck KGaA, Darmstadt, Germany), and incubated sequentially with primary and secondary antibodies after being blocked with 5% BSA. Finally, signals of proteins on the membrane were detected by chemiluminescence reagents (Millipore, Merck KGaA, Darmstadt, Germany) as per the manufacturer’s instructions and imaged with an Imaging System. The relative densitometry was calculated with Image J (1.50d version). The antibodies used for the Western blot analysis were FBL antibody (ab166630, Abcam plc, Cambridge Biomedical Campus, United Kingdom; 1:1000) and GAPDH antibody (2118s, Cell Signaling Technology, Inc., Danvers, MA, USA; 1:2000). Except for human specimens, Western blotting was performed in three independent replicates to confirm the reproducibility of the experiments.

### 2.10. RNA Extraction and RT-qPCR

Total RNA was extracted from cells or tissues using RNAiso Plus (Takara Bio Inc., Kusatsu, Shiga, Japan) in accordance with the manufacturer’s instructions. Reverse transcription was performed using PrimeScript RT Master Mix (Takara Bio Inc., Kusatsu, Shiga, Japan). qPCR was conducted using SYBR Premix Ex Taq II (Takara Bio Inc., Kusatsu, Shiga, Japan) and performed on the LightCycler^®^ 480 Real-Time PCR System (Roche Applied Science, Mannheim, Germany). GAPDH was used as a reference control, and the relative expression level of mRNA was calculated with 2^−ΔΔCt^ methods. Each experiment was performed in triplicate. The primer sequences used were as follows:

FBL Forward: 5′- GTCTTCATTTGTCGAGGAAAGGA-3′;

FBL Reverse: 5′- CTGGGTGAACTCCAAGGTG-3′;

GAPDH Forward: 5′- GGAGCGAGATCCCTCCAAAAT-3′;

GAPDH Reverse: 5′- GGCTGTTGTCATACTTCTCATGG-3′.

### 2.11. Immunohistochemistry

The paraffin sections were first dewaxed according to the standard procedures, and their endogenous peroxidase was inactivated in 3% H_2_O_2_. Then, they were heated up to 100 °C in Sodium Citrate-EDTA Antigen Retrieval Solution (P0085, Beyotime Institute of Biotechnology, Songjiang, Shanghai, China) under high pressure for 20 min for FBL antigen retrieval. Sections were incubated with FBL antibody (ab154806, Abcam plc, Cambridge Biomedical Campus, United Kingdom; 1:200) overnight at 4 °C and then incubated with HRP conjugated secondary antibody (DAKO, Agilent Technologies, Inc., Santa Clara, CA, USA) for 45 min at 37 °C. Finally, the antibody binding was visualized with diaminobenzidine (DAB) solutions, and sections were stained with hematoxylin for 3 min. The results of the staining were scored according to the semiquantitative scoring method. Immunoreactive score (0–12) was equal to the intensity of the staining (0 = not stained, 1 = low intensity, 2 = moderate intensity, 3 = high intensity) multiplied by the percentage of stained cells (0 = 0–5%, 1 = 6–25%, 2 = 26–50%, 3 = 51–75%, 4 = > 75%). The degree of staining was classified according to the final immunoreactive score: negative, 0 (−); weak, 1–4 (+); moderate, 5–8 (++); and strong, 9–12 (+++).

### 2.12. RNA Interference

FBL siRNA or negative control RNA at a final concentration of 75 nmol/L was transfected into cells with the lipofectamine 3000 transfection kit (Invitrogen, Thermo Fisher Scientific, Carlsbad, CA, USA) in accordance with the manufacturer’s instructions. At 48–72 h after transfection, cells were harvested for further experiments. The sequences of siRNA used were as follows:

Negative control (Sense: 5′-GTCTTCATTTGTCGAGGAAAGGA-3′, Antisense: 5′-CTGGGTGAACTCCAAGGTG-3′);

siFBL#1 (Sense: 5′-GACACUUUGUGAUUUCCAUTT-3′, Antisense: 5′-AUGGAAAUCACAAAGUGUCTT-3′);

siFBL#2 (Sense: 5′-CCTTGAGCCATATGAAAGATT-3′, Antisense: 5′-UCUUUCAUAUGGCUCAAGGTT-3′).

### 2.13. CCK8 Proliferation Assay

Cells transfected with siRNA for 48 h were seeded into a 96-well plate at a density of 1000 (Huh7) or 1500 (PLC/PRF/5) cells/well. After the planked cells grew adherent to the wall, CCK8 proliferation assay kits (Yeasen Biotechnology Co, Ltd., Pudong New Area, Shanghai, China) were used to test the cell proliferation at different time points (day 0,1,3,5) based on the manufacturer’s instructions. The absorbance at a wavelength of 450 nm was measured on a TS Microplate Reader (Tecan Group Ltd., Seestrasse, Männedorf, Switzerland). These experiments were performed in triplicate.

### 2.14. EdU Proliferation Assay

A total of 8 × 10^3^ huh7 cells or 1.5 × 10^4^ PLC/PRF/5 cells transfected with siRNA for 72 h were seeded into a 96-well plate. After 24 h, as the normal cell growth cycle was restored, an appropriate EdU solution was added to the 96-well plate with the cells mentioned above. After incubation for 2 h, cells were fixed with 4% paraformaldehyde for 15 min, and the EdU incorporated into the DNA of cells was detected by fluorescent antibodies in accordance with the manufacturer’s instructions. Finally, the proportion of proliferating cells was tested through a High Content Analysis on the ImageXpress Micro Confocal (Molecular Devices, San Jose, CA, USA). These experiments were performed in triplicate.

### 2.15. Colony Formation Assay

Huh7 or PLC/PRF/5 cells transfected with siRNA for 48 h were seeded into 6-well plates in complete growth medium. Initially, for Huh7, there were 1500 cells per well, and for PLC/PRF/5, there were 5000 cells per well. The culture medium was changed every 3 days. After 12 days, the cells were stained with 0.1% crystal violet after being fixed with 4% paraformaldehyde. The colony number was counted using ELISPOT Reader (AID GmbH, Penzberg, Germany). These experiments were performed in triplicate.

### 2.16. Sphere Formation Assay

A total of 500 cells transfected with siRNA for 72 h were seeded into low-adhesion 96-well plates in serum-free epithelial basal medium supplemented with B27 (50×) (GIBCO, Life Technologies Corporation, New York, NY, USA), 20 ng/mL EGF (PeproTech Inc., Cranbury, NJ, USA), and 20 ng/mL FGF (PeproTech Inc., Cranbury, NJ, USA) in each well. Fresh microsphere medium was regularly added for supplementary nutrients. Finally, we counted the spheres under a microscope after incubation for 7 days. These experiments were performed in triplicate.

### 2.17. Scratch Wound Healing Assay

When the cells transfected with siRNA for 72 h in a 6-well plate reached 95% confluence as a monolayer, we gently and slowly scratched the monolayer with a new 200 μL pipette tip across the center of the well to create a cross in each well. After scratching, serum-free medium was replenished into each well. Finally, we took photos of the scratches around the cross in each well under a microscope at 0 h (the time at which the scratching of the monolayer was completed) as well as 48 h later. The wound healing area of each scratch was quantitatively evaluated at different times using ImageJ. These experiments were performed in triplicate.

### 2.18. Transwell Migration and Invasion Assay

For the Transwell migration assay, 1 × 10^5^ huh7 cells or 2 × 10^5^ PLC/PRF/5 cells transfected with siRNA for 72 h were seeded with serum-free medium in the upper compartments of Transwell inserts (8 μm pore size, Corning, Glendale, AZ, USA), while medium containing 10% FBS was added to the lower bottom compartment. After 24 (huh7) or 36 h (PLC/PRF/5) of incubation, the inserts were taken out. Cells that remained in the upper chamber were gently removed with a cotton swab, and then cells on the lower chamber were fixed with 4% paraformaldehyde for 15 min and stained with 0.1% crystal violet for 20 min. Finally, we counted the number of cells on the lower chamber under a microscope. We randomly chose 5 different views and took the average number. These experiments were performed in triplicate.

For the Transwell invasion assay, 1 × 10^5^ huh7 cells or 2 × 10^5^ PLC/PRF/5 cells transfected with siRNA for 72 h were seeded with serum-free medium in the upper compartment of Transwell inserts (8 μm pore size, Corning, Glendale, AZ, USA) where the diluted Matrigel (diluted ratio = 1:9) was laid in advance, while medium containing 10% FBS was added to the lower bottom compartment. After 36 h of incubation, the inserts were taken out. Cells that remained on the upper chamber were gently removed with a cotton swab, and then cells on the lower chamber were fixed with 4% paraformaldehyde for 15 min and stained with 0.1% crystal violet for 20 min. Finally, the number of cells on the lower chamber was counted under a microscope. We randomly chose 3 different views and took the average number. These experiments were performed in triplicate.

### 2.19. Gene Set Enrichment Analysis

According to the median expression of FBL in hepatocellular carcinoma tissues in the TCGA and GSE14520 datasets, the samples were divided into high-expressing FBL samples and low-expressing FBL samples. Then, we imported the grouped sample data into the GSEA software (version 4.0.3) and selected “c7.all.v7.1.entrez.gmt” in the Molecular Signatures Database (MSig DB) on the GSEA website as the reference gene set to evaluate the impact of FBL on each reference set [14]. The default weighted enrichment statistical method was used to perform the Gene Set Enrichment Analysis (GSEA), and the number of random combinations was designed to be 1000. Gene sets that met the criteria of NES ≥ 1, NOM *p*-val < 0.05, and FDR q-val < 0.25 were defined as statistically significant enriched gene sets.

### 2.20. Establishment of the FBL Knockdown Stable Transfectant in Huh7

Huh7 cells were transfected with the FBL knockdown lentivirus and the corresponding negative control lentivirus (IGE Biotechnology Ltd., Guangzhou International Bio Island, Guangzhou, China) at a confluence of 50–70%, respectively. Multiplicity of infection (MOI) was set at 10. The fresh medium was replaced after transfection for 8 h. After 3 days, culture medium with 4 μg/mL puromycin was used to select successfully transfected cells. Finally, Western blotting was used to confirmed the efficacy of the infection 7 days later.

### 2.21. Xenograft Mouse Model

The 10 male nude mice (6–8 weeks old) were randomly divided into two groups. Then, 5 × 10^6^ Huh7 cells stably transfected with the negative control lentivirus or the FBL knockdown lentivirus were injected subcutaneously into mice with 100 μL PBS-Matrigel mixture (PBS: Matrigel = 1:1) respectively. Tumor sizes and volumes were measured regularly, and the tumor volumes were calculated as follows: tumor volume = width/2 × width × length. After 4–5 weeks, the mice were sacrificed, and the subcutaneous tumors were excised, imaged, and weighted. We strictly followed the guidelines for laboratory animal care of Sun Yat-Sen University, and the following conditions were regarded as the humane end point of this experiment: the maximum diameter of the subcutaneous tumor in nude mice exceeded 1.5 cm, or the weight loss of nude mice was greater than 10%, or there were obvious endangered manifestations in mice (such as significantly reduced food intake, significantly reduced activity, obvious respiratory depression, etc.).

### 2.22. Statistical Analysis

In this study, SPSS 24.0 was used for the statistical analysis, and the GraphPad Prism 7.0 software, Adobe Illustrator software, and R language were used for mapping. All in vitro experiments were performed in triplicate to confirm the reproducibility of the results obtained. All the experimental data are presented as the mean ± standard error (S.E.M.). Comparisons between two or more groups were statistically analyzed by the Student’s *t* test, Wilcoxon rank sum test, paired t-test, or random block design analysis of variance depending on the corresponding data type (Student’s *t* test: Western blotting analysis of FBL protein expression in hepatocellular carcinoma and adjacent liver tissues, tumor growth curve chart comparison, weight of gross tumors comparison; Wilcoxon rank sum test: FBL expression in hepatocellular carcinoma and adjacent liver tissues in TCGA and GEO; paired t-test: RT-qPCR analysis of FBL mRNA expression in hepatocellular carcinoma and adjacent liver tissues; random block design analysis: CCK8 proliferation assay, EdU Proliferation Assay, colony formation assay, sphere formation assay, scratch wound healing assay, Transwell migration and invasion assay between the control and two experimental groups). Four-grid table data were statistically analyzed by the χ^2^ test. *p* < 0.05 was considered statistically significant. Log-rank test was used for overall survival and disease-free survival without curved intersection. Peto-Peto’s modified survival estimate test was used for overall survival and disease-free survival with curved intersection. Pearson correlation test was performed to analyze the correlation amongst FBL and other genes.

## 3. Results

### 3.1. Coexpression Network Construction and Module Identification

**Figure 2:**
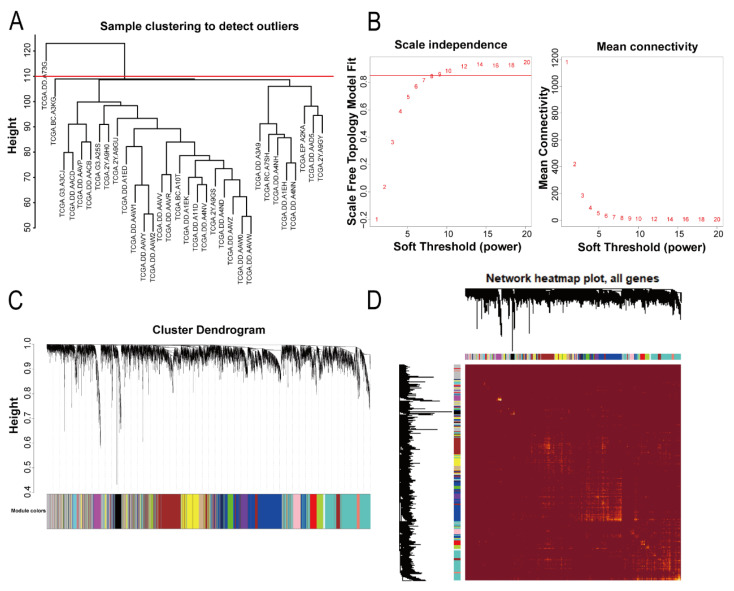
Coexpression network construction and module identification. (**A**) As shown by the hierarchical clustering graphs of 18 samples of lung metastases after hepatectomy and 14 samples without recurrence and metastasis within 5 years after hepatectomy in TCGA, one outlier was removed. (**B**) A suitable soft threshold β value of 9 was selected. (**C**) A gene clustering dendrogram based on a topological overlap matrix (TOM) network of genes was constructed with the top 5000 median absolute deviation (MAD) size. The color bands represent the modules classified by the hybrid dynamic shearing tree. Seventeen modules were identified. (**D**) The heatmap plot shows the topological overlap matrix (TOM) for all genes and the interactive relations among the 17 modules. The light color represents a high degree of overlap. The left side is the gene dendrogram and the top side is the module assignment.

Hierarchical clustering with the Euclidean distance was used to determine whether there were sample outliers. As shown in Figure 2A, one sample (TCGA.DD.A73G) was found to deviate significantly from the group, so it was removed from the analysis. We constructed a weighted gene coexpression network for the remaining samples. As shown in Figure 2B, the optimal soft threshold β value was 9, which could make the gene distribution conform to the scale-free network. According to the hybrid hierarchical clustering and dynamic cut tree method, 17 modules were identified with a different color for each module (Figure 2C). Afterward, we constructed a heatmap of the network to visualize the topological overlap matrix (TOM) among all the genes and analyze the interactive relations among the 17 modules (Figure 2D).

### 3.2. Identification of Significant Modules and Hub Genes

**Figure 3:**
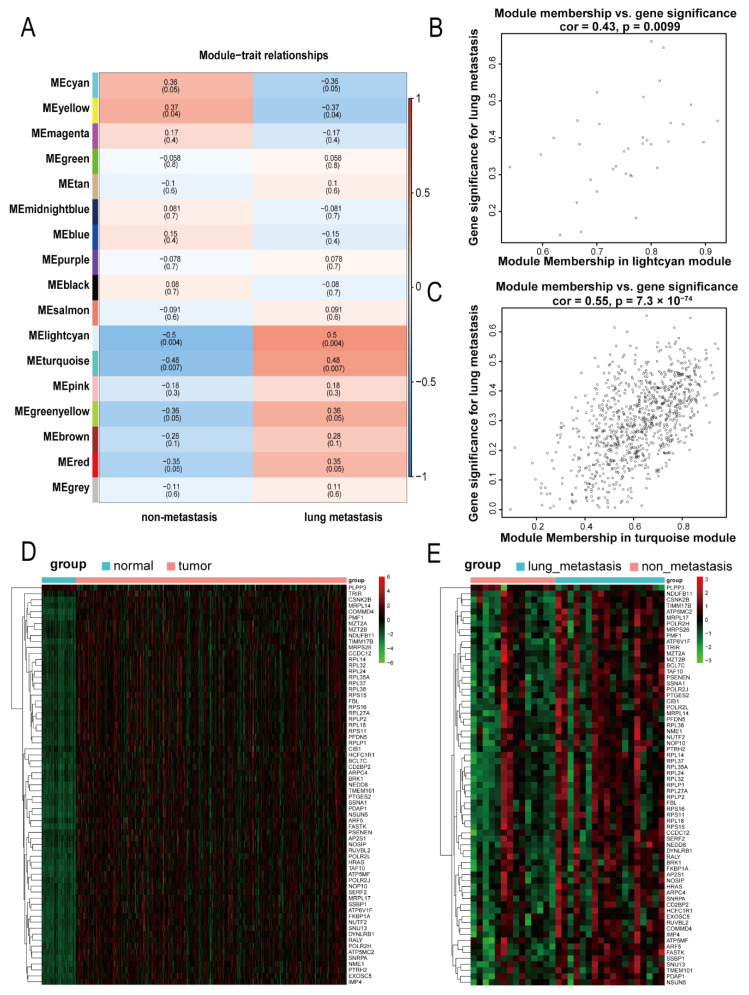
Identification of significant modules and hub genes. (**A**) The heatmap shows the correlations between the modules and the traits of interest (lung metastasis or without metastasis). (**B**,**C**) The scatter plots show the correlations between the module membership (MM) and the gene significance (GS) in the light cyan module and turquoise module related to lung metastasis of hepatocellular carcinoma. (**D**) The heatmap displays the expression of 67 hub genes related to lung metastasis in hepatocellular carcinoma tissues and normal liver tissues in the TCGA. (**E**) The heatmap displays the expression of 67 hub genes related to lung metastasis in hepatocellular carcinoma tissues with lung and without metastasis in the TCGA.

A correlation analysis was performed for the 17 modules obtained and the traits of interest (lung metastasis) to look for significant associations. As shown in Figure 3A, the light cyan module and the turquoise module with *p* value < 0.01 were identified as significant modules. The correlation coefficients between the two modules mentioned above and lung metastasis were 0.5 and 0.48, respectively. These values were higher than the absolute values of the correlation coefficients of other modules, indicating that these two modules had the closest relationship with lung metastasis. We further analyzed the internal genes of the two modules mentioned above. As shown in Figure 3B,C, the correlation coefficient between GS and MM in the light cyan module was 0.43, and that for the turquoise module was 0.55, indicating that the two modules were suitable for identifying genes related to lung metastasis. The genes with GS >0.4 and MM >0.8 in significant modules were identified as hub genes. In the light cyan module, there were 8 hub genes; in the turquoise module, there were 61 hub genes. After taking the union of the two, we obtained 67 hub genes related to lung metastasis (Table S1). Next, we used the transcriptome sequencing data in the TCGA to show the difference in expression of these 67 genes related to lung metastasis in hepatocellular carcinoma tissues and normal liver tissues as well as that between hepatocellular carcinoma tissues with and without lung metastasis. As shown in Figure 3D,E, compared with normal liver tissues, 67 genes related to lung metastasis were highly expressed in hepatocellular carcinoma tissues (Table S2), and compared with hepatocellular carcinoma tissues without metastasis, most of 67 genes related to lung metastasis were also upregulated in hepatocellular carcinoma tissues with lung metastasis in TCGA dataset (Table S3).

### 3.3. Gene Ontology Annotations and Kyoto Encyclopedia of Genes and Genomes Pathway Analysis of 67 Hub Genes Related to Lung Metastasis

**Figure 4:**
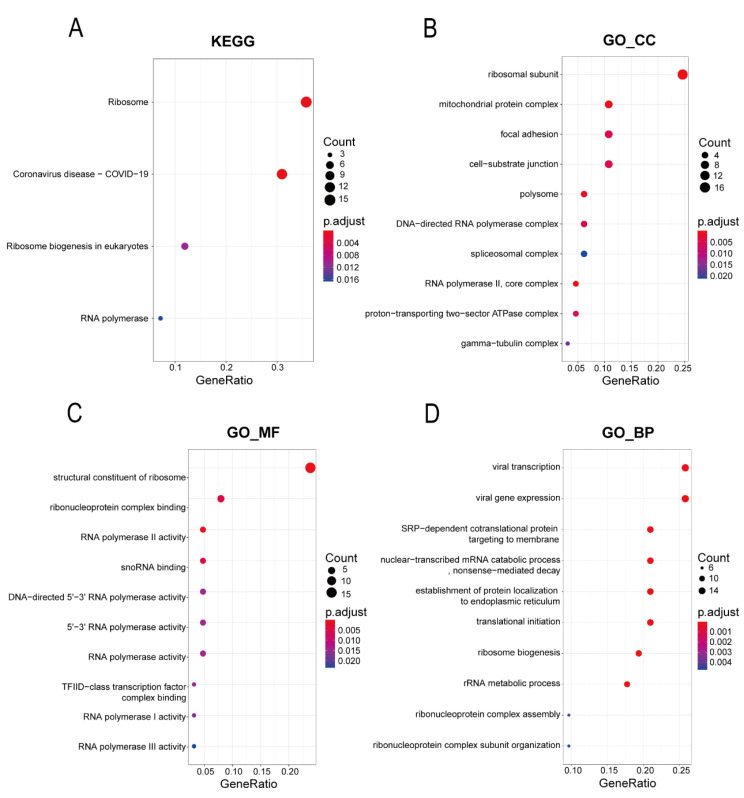
Gene Ontology annotations and Kyoto Encyclopedia of Genes and Genomes pathway analysis of the 67 hub genes related to lung metastasis. (**A**) The results of the KEGG pathway analysis. (**B**) The results for cellular component enrichment in GO. (**C**) The results for molecular function enrichment in GO. (**D**) The results for biological process enrichment in GO.

The KEGG pathway enrichment analysis was performed on 67 hub genes related to lung metastasis. As shown in Figure 4A, a total of four pathways were found to be enriched, and these were associated with the ribosome and RNA polymerase. The GO analysis results showed that the 67 hub genes were mainly distributed among items related to the ribosome, mitochondria, and RNA transcription for cellular components (CCs), such as the ribosomal subunit, mitochondrial protein complex, and RNA polymerase II-core complex (Figure 4B). They were mainly concentrated in items related to the ribosome composition and RNA polymerase activity for molecular functions (MFs), such as the structural constituent of the ribosome, RNA polymerase II activity, and snoRNA binding (Figure 4C). They were enriched in items related to ribosome synthesis and protein translation processes for biological processes (BP), such as SRP-dependent co-translational protein targeting of the membrane, ribosome biogenesis, and rRNA metabolic processes (Figure 4D). This indicates that ribosome and rRNA-related pathways might play important roles in lung metastasis associated with hepatocellular carcinoma.

### 3.4. Protein–Protein Interaction Network Analysis of 67 Hub Genes Related to Lung Metastasis

**Figure 5:**
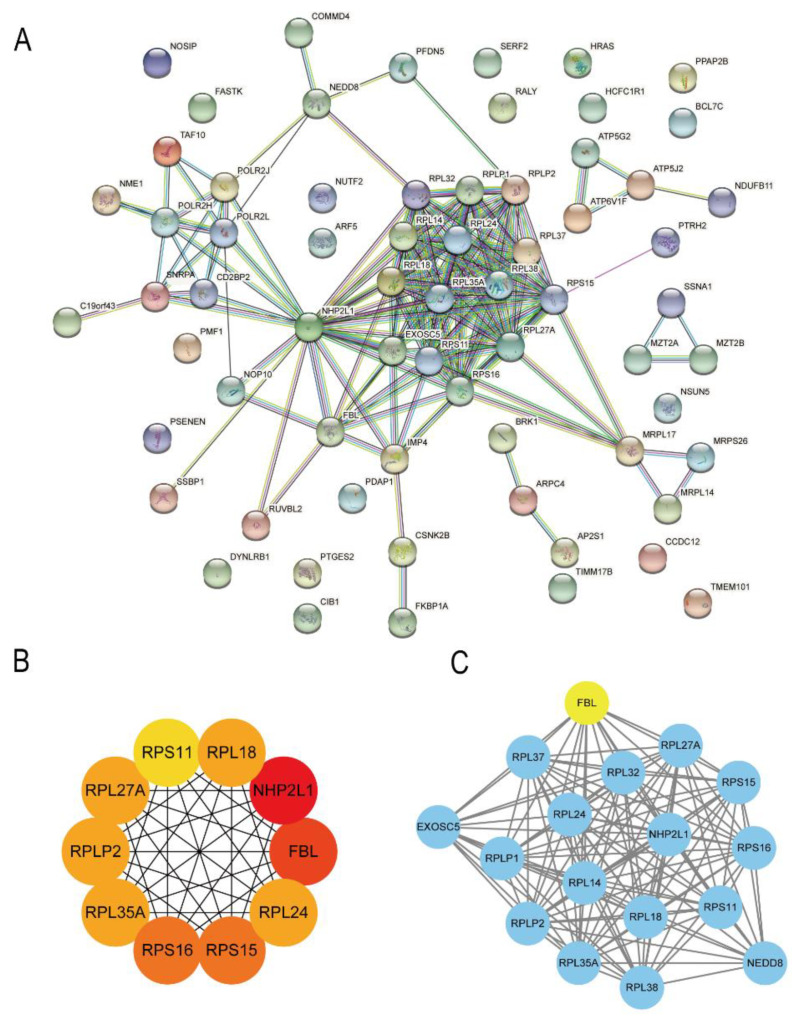
Protein–Protein Interaction network analysis of the 67 hub genes related to lung metastasis. (**A**) The PPI network between the 67 hub genes related to lung metastasis based on the STRING database. (**B**) The top 10 genes with high degree scores in the cytoHubba analysis. The deep color represents high scores. (**C**) The core subnetwork in the MCODE analysis. The gene marked in yellow is the seed gene for the network.

After entering the 67 genes related to lung metastasis in the STRING database, 67 nodes and 246 edge PPI networks were obtained (Figure 5A). In order to simplify the PPI network to find the most critical core genes, the cytoHubba plug-in from the Cytoscape software was used to obtain the degree score of each gene. The top 10 genes were ribosomal protein-coding genes or genes related to ribosome production, including *NHP2L1*, *FBL*, *RPS15*, *RPS16*, *RPL18*, *RPL35A*, *RPLP2*, *RPL27A*, *RPL24*, and *RPS11* (Figure 5B). In addition, we obtained the core subnetwork through the MCODE plug-in of the Cytoscape software. As shown in Figure 4C, we found that FBL was the seed gene of the core subnetworks.

### 3.5. FBL Is Highly Expressed in Hepatocellular Carcinoma, and Its High Expression Is Closely Related to Poor Prognosis and Lung Metastasis in Hepatocellular Carcinoma Patients

**Figure 6:**
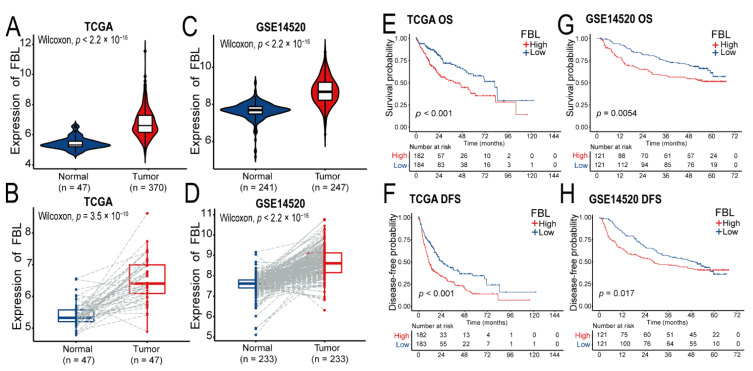
FBL is highly expressed in hepatocellular carcinoma patients, and the prognosis of hepatocellular carcinoma patients with high FBL expression was found to be worse according to data from the TCGA and GEO databases. (**A**,**B**) FBL expression in hepatocellular carcinoma and adjacent liver tissues in TCGA; (B) shows a comparative analysis of paired samples. (**C**,**D**) FBL expression in hepatocellular carcinoma and adjacent liver tissues in GSE14520; (**D**) shows a comparative analysis of paired samples. (**E**,**F**) Overall survival (OS) and disease-free survival (DFS) between FBL high expression and low expression groups of hepatocellular carcinoma patients in the TCGA. (**G**,**H**) Overall survival (OS) and disease-free survival (DFS) between FBL high expression and low expression groups of hepatocellular carcinoma patients in the GSE14520. (**A**–**D**) Wilcoxon rank sum test. (**E**–**H**) Peto-Peto’s modified survival estimate test.

According to the results of the Protein–Protein Interaction network analysis, we selected the ribosome-related gene FBL, which might play an important role for in-depth studies. In order to determine whether FBL is highly expressed in hepatocellular carcinoma, we analyzed FBL expression in samples of hepatocellular carcinoma tissue and normal liver tissue adjacent to the cancer in the TCGA and GSE14520. As shown in Figure 6A,C, the concentration of FBL mRNA in hepatocellular carcinoma tissues was significantly higher than that in normal liver tissues (*p* < 0.001). To improve the comparability, we further carried out a paired analysis of the tumor tissues and adjacent normal tissues from the above datasets, and the differences were found to be statistically significant (Figure 6B,D). Moreover, in order to analyze the correlation between FBL expression and clinical prognosis in patients with hepatocellular carcinoma, we divided the two cohorts (TCGA and GSE14520) into a high-expressing FBL group and a low-expressing FBL group according to the median of FBL expression. The results of the survival analysis showed that high FBL expression was positively correlated with a shorter overall survival (OS) (Figure 6E,G) and shorter disease-free survival (DFS) (Figure 6F,H) in hepatocellular carcinoma patients, and the differences were found to be statistically significant.

**Figure 7:**
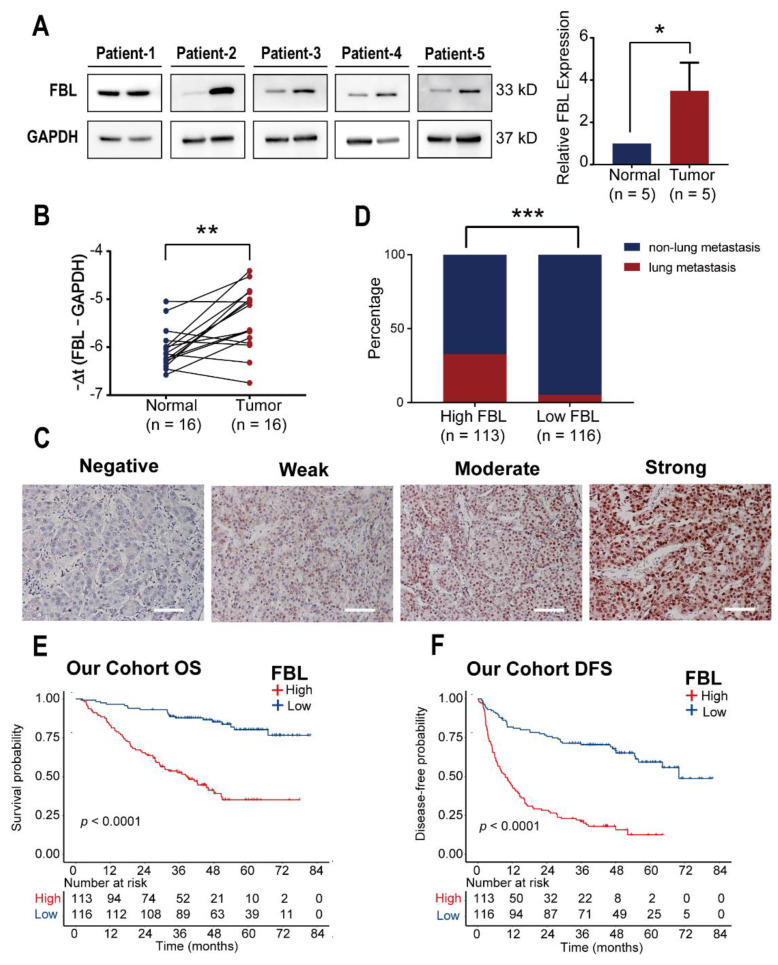
FBL is highly expressed in hepatocellular carcinoma, and high expression of FBL is positively correlated with the occurrence of lung metastasis, indicating a poor prognosis in our cohort. (**A**) Western blotting analysis of FBL protein expression in hepatocellular carcinoma and adjacent liver tissues. GAPDH was used as an internal reference. In each band, normal tissue is shown on the left, and tumor tissue is shown on the right. (**B**) RT-qPCR analysis of FBL mRNA expression in hepatocellular carcinoma tissue and adjacent liver tissue. The ordinate is −Δt (the number of PCR cycles of FBL minus the number of PCR cycles of the internal control GAPDH). (**C**) Representative images of hepatocellular carcinoma paraffin specimens stained by the immunohistochemical method that display the different expression levels of FBL. (**D**) Comparison of the incidence of lung metastasis in hepatocellular carcinoma patients with high or low expression of FBL. (E, F) Overall survival (OS) and disease-free survival (DFS) between FBL high expression and low expression groups of hepatocellular carcinoma patients in our cohort. * *p* < 0.05, ** *p* < 0.01, *** *p* < 0.001. (**A**) Student’s *t* test. (**B**) paired *t*-test. (**D**) χ^2^ test. (**E**,**F**) Log-rank test.

In order to verify the above findings, we first detected the expression of the FBL protein in five pairs of matched hepatocellular carcinoma tissues and adjacent normal liver tissues by Western blot analysis (Figure 7A). The expression of FBL mRNA was detected by RT-qPCR in 16 pairs of matched hepatocellular carcinoma tissues and adjacent normal liver tissues (Figure 7B). The results showed that, compared with adjacent normal liver tissues, the expression of FBL in terms of the mRNA level and protein level in hepatocellular carcinoma tissues was upregulated, and the differences were statistically significant. Meanwhile, immunohistochemistry was used to detect FBL expression in 229 patients with hepatocellular carcinoma in our hospital. According to the immunohistochemistry results, we divided hepatocellular carcinoma patients into a low FBL expression group (Figure 7C, Negative and Weak) and a high FBL expression group (Figure 7C, Moderate and Strong), and the clinical data were sorted and analyzed according to the grouping (Table 1). We found that, compared with hepatocellular carcinoma patients with low FBL expression, hepatocellular carcinoma patients with high FBL expression had a higher probability of experiencing lung metastasis (Figure 7D). In addition, consistent with the results of the survival analysis in the TCGA and GSE14520, the overall survival of patients with high FBL expression was shorter (Figure 7E), and disease-free survival was also shorter (Figure 7F), and the differences were statistically significant. Moreover, as shown in Table 1, the high expression of FBL in hepatocellular carcinoma patients was closely related to the serum alpha-fetoprotein level, the number of tumors, the pathological grade, vascular invasion, and TNM staging.

### 3.6. Knockdown of FBL Suppresses Proliferation and Stemness in HCC Cell Lines

**Figure 8:**
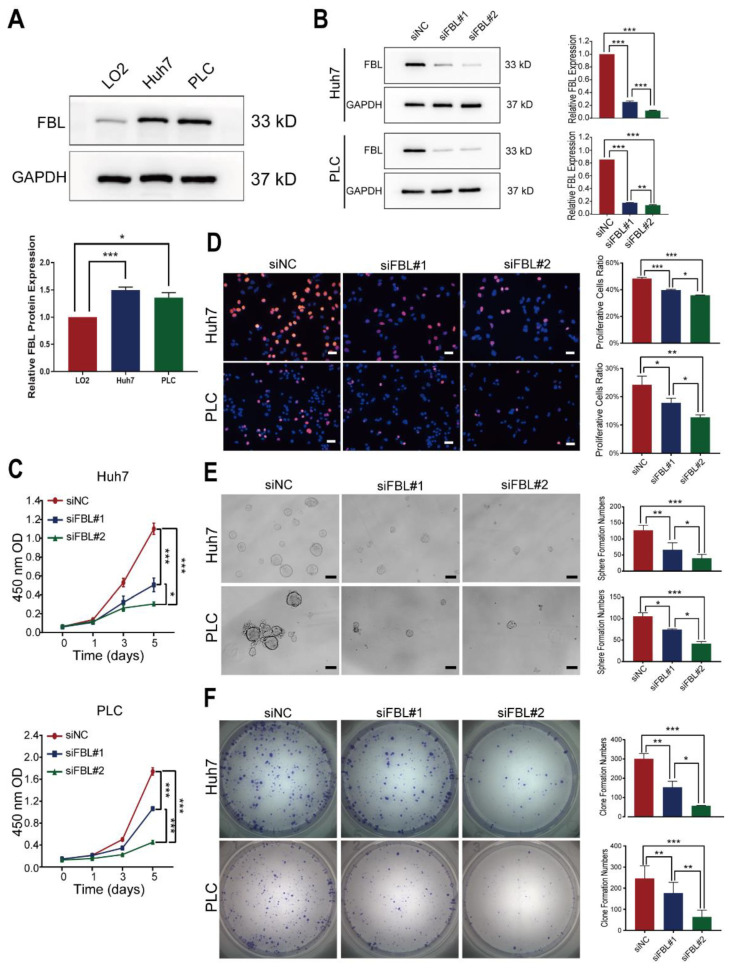
Knockdown of FBL suppresses proliferation and stemness in HCC cell lines. (**A**) The protein level of FBL in an immortalized liver cell line (LO2) and HCC cell lines (Huh7 and PLC/PRF/5) were measured by Western blotting. GAPDH was used as a loading control. (**B**) The knockdown efficiency of two siRNA sequences (siFBL#1 and siFBL#2) on FBL in Huh7 and PLC/PRF/5 cells was detected by Western blot analysis. siNC is the control group. (**C**) After 48 h post-siRNA transfection, the CCK8 kit was used to detect the effect of FBL knockdown on the proliferation of Huh7 and PLC/PRF/5 cells after 0, 1, 3, and 5 days. The absorbance value at 450 nm detected by the microplate reader was 450 nm OD. (**D**) After 48 h post-siRNA transfection, the EdU kit was used to detect the effect of FBL knockdown on the DNA synthesis ability of Huh7 and PLC/PRF/5 cells. Scale bar: 50 μm. (**E**) The effect of FBL knockdown on the stemness of Huh7 and PLC/PRF/5 cells was verified by sphere formation assay. Scale bar: 100 μm. (**F**) The effect of FBL knockdown on the stemness of Huh7 and PLC/PRF/5 cells was verified by clone formation assay. These experiments were performed in triplicate. * *p* < 0.05, ** *p* < 0.01, *** *p* < 0.001. (**A**) Student’s *t* test. (**B**–**E**) Random block design analysis.

As shown in Figure 8A, the expression of FBL in HCC cell lines (Huh7 and PLC/PRF/5) were higher than that in immortalized liver cell line (LO2). The huh7 and PLC/PRF/5 cell lines were chosen to establish the FBL knockdown models through siRNA in our study. The knockdown efficiency of FBL was verified by Western blotting analysis, indicating that the silencing efficiency of two siRNA sequences was >70%. The siFBL#2 sequence was found to have a higher silencing efficiency than the siFBL#1 sequence (Figure 8B). To determine the effect of FBL knockdown in the proliferation of cells mentioned above, Cell-counting kit-8 (CCK-8) assays were performed, and the results showed that the silencing of FBL inhibited the proliferation of HCC cells compared with the control (Figure 8C). Furthermore, EdU assays also revealed that the silencing of FBL could inhibit the proliferation of HCC cells (Figure 8D). As we can see in Figure 8C, DNA synthesis was significantly reduced in FBL-silenced groups (red dots represented cells undergoing DNA synthesis). It was reported that FBL played an important role in promoting pluripotency in embryonic stem cells, but the role of FBL in the stemness of HCC cells is unknown [15]. Thus, we performed sphere formation and colony formation assays to compare the level of stemness between the FBL-silenced group and the control group. The results indicate that the silencing of FBL could remarkably reduce sphere formation (Figure 8E) as well as colony formation (Figure 8F) in HCC cell lines.

### 3.7. Knockdown of FBL Suppresses Migration and Invasion in HCC Cell Lines

**Figure 9:**
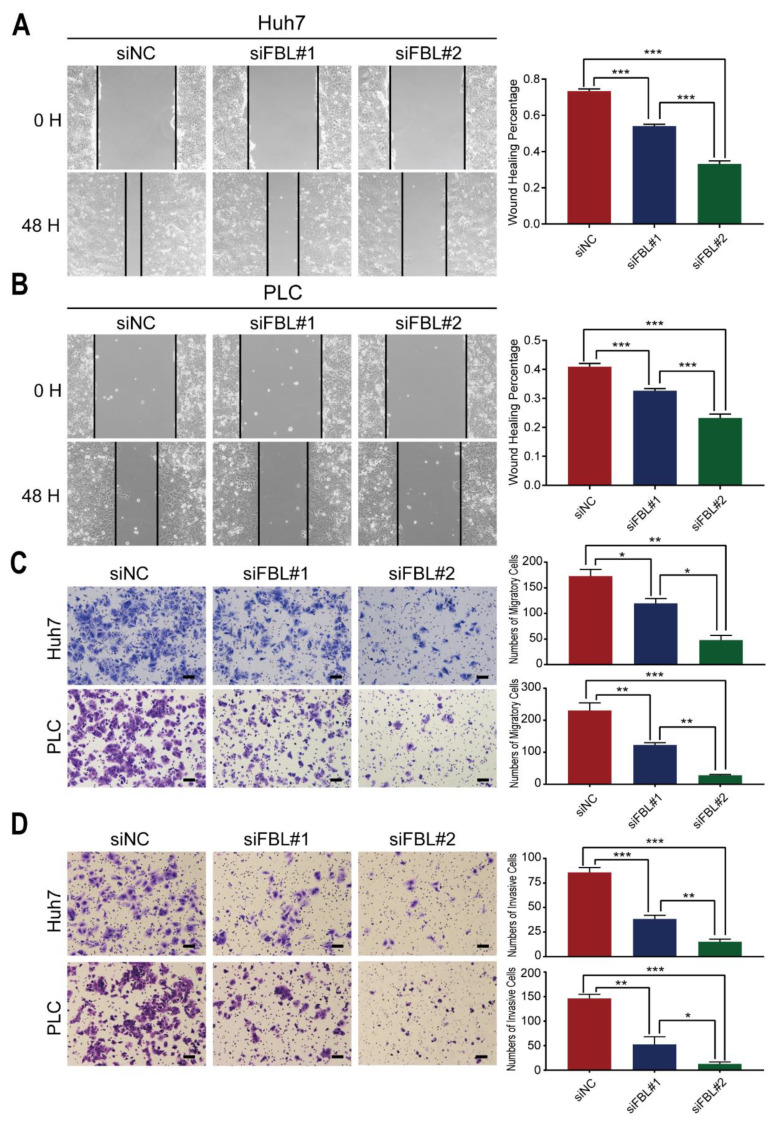
Knockdown of FBL suppresses migration and invasion in HCC cell lines. (**A**, **B**) The effect of FBL knockdown on the migration ability of Huh7 and PLC/PRF/5 cells was verified by a scratch wound healing assay. Forty-eight hours after scratching, the differences in the degree of wound healing between the two knockdown FBL experimental groups (siFBL#1 and siFBL#2) and the control group (siNC) transfected with control siRNA were detected. (**C**) The effect of FBL knockdown on the migration ability of Huh7 and PLC/PRF/5 cells was verified by the Transwell migration assay. Scale bar: 100 μm. (**D**) The effect of FBL knockdown on the invasion ability of Huh7 and PLC/PRF/5 cells was verified by the Transwell invasion assay. Scale bar: 100 μm. These experiments were performed in triplicate. * *p* < 0.05, ** *p* < 0.01, *** *p* < 0.001. (**A**–**D**) random block design analysis.

Migration and invasion capacities are characteristics of highly malignant cells and are important causes of tumor metastasis. The results mentioned above show that FBL is associated with metastasis, so we were interested to determine whether FBL could affect the migration and invasion of hepatoma cells. To investigate the effect of FBL in migration, we conducted both wound healing assays and Transwell migration assays. The results showed that FBL silencing evidently slowed the speed of wound healing (Figure 9A,B) and reduced the number of migratory cells in the Transwell migration assay (Figure 9C). In addition, Transwell invasion assays were carried out to test the invasion capacity after FBL silencing, and we found that FBL knockdown greatly weakened the capacity of invasion in HCC cell lines (Figure 9D).

### 3.8. FBL Might Be Involved in Multiple Signaling Pathways in Hepatocellular Carcinoma

**Figure 10:**
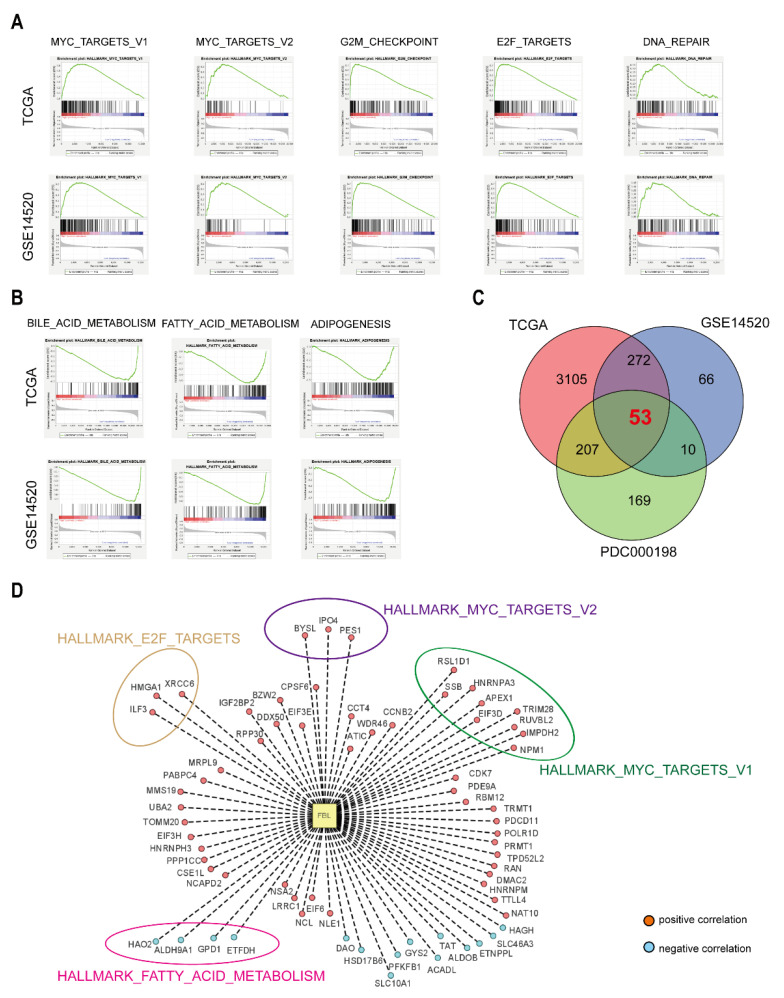
Exploring possible pathways involved in FBL in hepatocellular carcinoma. (**A**) Gene sets simultaneously enriched in the TCGA and GSE14520 datasets in the FBL high expression group. (**B**) Gene sets simultaneously enriched in the TCGA and GSE14520 datasets in the FBL low expression group. (**C**) Venn diagram showed 53 genes correlated to FBL among TCGA, GSE14520, PDC000198 with absolute coefficient > 0.4. (**D**) A total of 53 genes correlated to FBL among TCGA, GSE14520, and PDC000198, parts of which were involved in MYC_TARGETS_V1, MYC_TARGETS_V2, E2F_TARGETS, and FATTY_ACID_METABOLISM based on GSEA gene sets. Red plots: positive correlation with FBL. Blue plots: negative correlation with FBL.

The hepatocellular carcinoma samples in the TCGA and GSE14520 databases were classified into FBL high expression or FBL low expression groups according to the median FBL expression. We performed a gene set enrichment analysis (GSEA) with the MSigDB hallmark gene set on the FBL high expression and low expression groups from the TCGA and GSE14520 datasets. As shown in Figure 10A, there were five gene sets in the FBL high expression group that were simultaneously enriched in the above two datasets, namely the MYC_TARGETS_V1, MYC_TARGETS_V2, G2M_CHECKPOINT, E2F_TARGETS, and DNA_REPAIR gene sets. According to previous research, all of the gene sets mentioned above are closely associated with cell proliferation, and the MYC gene and E2F gene family were also found to be closely related to the stemness and metastasis of tumors, which suggests that FBL might participate in the regulation of these pathways and affect the proliferation, stemness, migration, and invasion of hepatocellular carcinoma. In addition, as shown in Figure 10B, three gene sets in the FBL low expression group were simultaneously enriched in the above two datasets. These three gene sets were BILE_ACID_METABOLISM, FATTY_ACID_METABOLISM, and ADIPOGENESIS, which suggests that FBL might be related to bile acid metabolism and fatty acid metabolism in hepatocellular carcinoma.

To further explore molecules responsible for FBL-induced metastasis, Pearson correlation analysis was used to find the genes correlated to FBL in TCGA, GSE14520, and PDC000198. As shown in Figure 10C, amongst three databases mentioned above, there were 53 genes correlated to FBL with absolute coefficient >0.4 (Table S4). Among 53 genes, *BYSL*, *IPO4*, *PES1*, *SSB*, *HNRNPA3*, *RSL1D1*, *APEX1*, *NPM1*, *EIF3D*, *RUVBL2*, *TRIM28*, and *IMPDH2* were parts of MYC signaling pathway; *ILF3*, *HMGA1*, and *XRCC6* were parts of E2F signaling pathway; and *ETFDH*, *GPD1*, *HAO2*, and *ALDH9A1* were parts of fatty acid metabolism (Figure 10D). It is reasonable to infer that these molecules are responsible for FBL-induced metastasis.

### 3.9. Knockdown of FBL Suppresses Hepatocellular Carcinoma Cell Growth In Vivo

**Figure 11:**
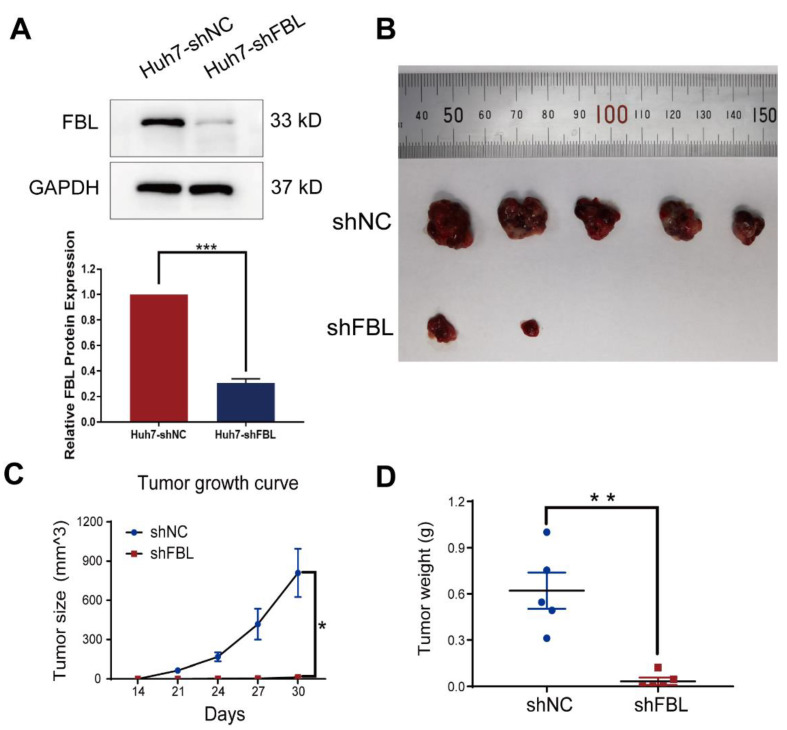
Knockdown of FBL suppresses hepatocellular carcinoma cell growth in vivo. (**A**) The knockdown efficiency of FBL knockdown lentivirus (shFBL) on FBL in Huh7 cells was detected by Western blotting analysis. shNC is the control group. (**B**) Gross tumors excised from the nude mice subcutaneously injected with Huh7 cells stably transfected with shNC or shFBL.. The smallest scale division of the ruler is 0.1 mm. (**C**) Tumor growth curve chart, tumor sizes in nude mice subcutaneously injected with Huh7 cells stably transfected with shNC or shFBL were measured at days 14, 21, 24, 27, and 30. (**D**) Weights of gross tumors excised from the nude mice subcutaneously injected with Huh7 cells stably transfected with shNC or shFBL. * *p* < 0.05, ** *p* < 0.01, *** *p* < 0.001. (**A**,**C**,**D**) Student’s *t* test.

It has been reported that the knockdown of FBL is a negative factor for tumor growth in many cancers, such as breast cancer and prostate cancer [16,17]. However, the role of FBL in hepatocellular carcinoma cell growth in vivo is unclear. Therefore, we subcutaneously injected Huh7 cells stably transfected with the negative control lentivirus (shNC) and the FBL knockdown lentivirus (shFBL) into nude mice, respectively. As shown in Figure 11A, the knockdown efficiency of FBL was verified by Western blotting analysis, indicating that the silencing efficiency of FBL knockdown lentivirus was significant. The results showed that the knockdown of FBL significantly inhibited tumor growth and tumorigenesis compared with the control group (Figure 11B–D).

## 4. Discussion

Lung metastasis is an important factor leading to poor prognosis in hepatocellular carcinoma patients. In-depth research on the molecular mechanism associated with lung metastasis in hepatocellular carcinoma will help us to predict the prognosis of hepatocellular carcinoma more accurately and provide a scientific basis for its prevention and treatment.

Many previous studies found that the phenomenon of the over-synthesis of ribosomes exists in a variety of tumors, and it plays an important role in the occurrence and development of tumors [18,19]. As the “factories” of protein synthesis, ribosomes are mainly composed of ribosomal proteins and ribosomal RNA. Ribosomal proteins are divided into large ribosomal proteins (RPL) and small ribosomal proteins (RPS). Previous studies showed that ribosomal proteins mainly participate in the process of protein synthesis. However, with the deepening of research, researchers have found that ribosomal proteins are also involved in the activation of proto-oncogenes or tumor suppressor genes that regulate the cell cycle through multiple signal pathways to promote the growth and proliferation of cells [20]. In addition, a variety of ribosomal proteins have been reported to be associated with tumor metastasis. For instance, the high expression of *RPS6* in lung cancer is significantly related to the increased risk of early metastasis [21]. Knocking down *RPL39* could significantly inhibit the occurrence of lung metastasis of breast cancer in immunodeficiency mouse models, and most breast cancer patients with lung metastasis have acquired mutations of *RPL39* [22]. Knocking out *RPL15* using CRISPR technology significantly weakened lung metastasis in breast cancer circulating tumor cells in mouse models, and with *RPL15* overexpression, breast cancer circulating tumor cells had stronger lung and ovarian metastasis ability [23]. However, the tumor metastasis mechanism affected by the ribosomal protein has not been clearly reported in the past research. According to the clues provided in the existing research, there are two types of mechanisms: the first is that a variety of ribosomal proteins could promote the composition and activity of ribosomes, which might affect the translation efficiency of specific mRNA types [24]; the other is that it might affect the activation or inhibition of signal pathways through the binding of specific proteins, thereby affecting tumor metastasis [20]. Our study found that ribosomal protein genes account for a large proportion of the modules related to lung metastasis analyzed by WGCNA, such as *RPS15*, *RPS16*, *RPL18*, *RPL35A*, *RPLP2*, *RPL27A*, *RPL24*, and *RPS11*. These ribosomal proteins are likely to play important roles in lung metastasis associated with hepatocellular carcinoma, but the related mechanisms need further verification through in vivo and in vitro experiments.

Fibrillarin (FBL) is a 2′-O methyltransferase that is involved in ribosomal biogenesis [25]. It is the main constituent protein of box C/D snoRNP (small nucleolar ribonucleoprotein particles). It is upregulated in prostate cancer, breast cancer, and other tumors, and it can promote tumor cell growth, proliferation, and drug resistance [26,27,28]. Additionally, recent research suggested that increased Fibrillarin expression is related to poor tumor prognosis in hepatocellular carcinoma [29]. However, few studies have investigated whether *FBL* can promote the progression of hepatocellular carcinoma. Our research preliminarily proves that *FBL* could affect the proliferation, stemness, migration, and invasion of hepatocellular carcinoma cells, which might be involved in the activation of MYC and E2F signaling. In previous research, it was shown that the tumor suppressor gene *TP53* and the proto-oncogene *MYC* are upstream regulators of *FBL* [16,27]. In breast cancer cells, *TP53* can inhibit the expression of *FBL*. In prostate cancer, however, the expression of *FBL* can be upregulated by *MYC*. As for hepatocellular carcinoma, further study is required to determine which gene plays the main regulatory role in *FBL* expression. Regarding the downstream regulation of *FBL*, in addition to participating in the regulation of ribosome synthesis, the latest research shows that *FBL* could directly modify specific types of mRNA by 2′-O methylation, thereby inhibiting its translation efficiency and achieving the regulation of specific gene expression, which suggests that the effect of *FBL* on the biological function of hepatocellular carcinoma may also play a role in this regard [30]. Besides, our study demonstrated that genes related to MYC and E2F signaling pathway, such as *BYSL*, *IPO4*, *PES1*, *SSB*, *HNRNPA3*, *RSL1D1*, *APEX1*, *NPM1*, *EIF3D*, *RUVBL2*, *TRIM28*, *IMPDH2*, *ILF3*, *HMGA1*, and *XRCC6*, were strongly positively correlated to FBL, which means that they may be the downstream molecules responsible for *FBL*-induced lung metastasis.

In general, our research provides ideas for the prediction of the prognosis of hepatocellular carcinoma patients as well as a scientific basis for research on the mechanisms associated with lung metastasis occurrence in hepatocellular carcinoma.

## 5. Conclusions

Ribosome-biogenesis-related proteins may play an important role in lung metastasis associated with hepatocellular carcinoma. As a hub gene in ribosome-biogenesis-related proteins, *FBL*, which is closely related to the poor prognosis of HCC patients, can promote the growth and metastasis of hepatocellular carcinoma.

## Figures and Tables

**Table 1:** Correlation between FBL expression and clinicopathological features.

Variables		All Cases	FBL		*p*-Value
			Low Expression	High Expression	
Gender	Male	188	100	88	0.100
	Female	41	16	25	
Age (years)	<65	196	98	98	0.429
	≥65	33	18	15	
Drinking	NO	184	92	92	0.688
	YES	45	24	21	
HBV	Negative	40	21	19	0.797
	Positive	189	95	94	
Cirrhosis	NO	61	32	29	0.742
	YES	168	84	84	
AFP (ng/mL)	<300	135	78	57	0.01 *
	≥300	94	38	56	
Tumor encapsulation	NO	184	90	94	0.286
	YES	45	26	19	
Tumorous number	Single	183	101	82	0.006 **
	Multiple	46	15	31	
Differentiation grade	I-II	132	76	56	0.021 *
	III-IV	97	40	57	
Cancer Embolus	NO	134	63	71	0.191
	YES	95	53	42	
Vascular invasion	NO	171	98	73	<0.001 ***
	YES	58	18	40	
TNM stage	I	90	57	33	0.002 **
	II-III-IV	139	59	80	
Postoperative pulmonary metastasis	NO	186	110	76	<0.001 ***
	YES	43	6	37	

HBV, hepatitis B virus; AFP, Alpha-fetoprotein; TNM, tumor-node-metastasis. * *p* < 0.05, ** *p* < 0.01, *** *p* < 0.001. χ^2^ test is used here.

## Data Availability

Publicly available datasets were analyzed in this study. These data can be found here: 1. [https://portal.gdc.cancer.gov/projects/, Project ID: TCGA-LIHC]. 2. [https://www.ncbi.nlm.nih.gov/geo/, GEO accession: GSE14520]. 3. [https://pdc.cancer.gov/pdc/, Study Identifier: PDC000198].

## Supplementary Materials

The following supporting information can be downloaded at: https://www.mdpi.com/article/10.3390/bioengineering9080396/s1, Table S1: The hub genes in Turquoise module and Light cyan module and 67 hub genes related to lung metastasis coming from unit of Turquoise module and Light cyan module; Table S2: The expression of 67 genes between normal liver tissues and hepatocellular carcinoma tissues; Table S3: The expression of 67 genes between hepatocellular carcinoma tissues without metastasis and hepatocellular carcinoma tissues with lung metastasis; Table S4: 53 genes correlated to FBL with absolute coefficient > 0.4 in TCGA, GSE14520 and PDC000198.

## Abbreviations

CCK8	Cell Counting Kit-8
FBL	Fibrillarin
GAPDH	glyceraldehyde-3-phosphate dehydrogenase
GEO	Gene Expression Omnibus
GO	Gene Ontology
GSEA	Gene Set Enrichment Analysis
HCC	hepatocellular carcinoma
KEGG	Kyoto Encyclopedia of Genes and Genomes
PPI	Protein–Protein Interaction
RT-qPCR	real-time quantitative PCR
STRING	Search Tool for the Retrieval of Interacting Genes
TCGA	The Cancer Genome Atlas
WGCNA	weighted gene coexpression network analysis
