# Supplementary material for: Bioinformatic Analysis and In Vitro and In Vivo Experiments Reveal That Fibrillarin Participates in the Promotion of Lung Metastasis in Hepatocellular Carcinoma
Source: Bioengineering (Basel). 2022 Aug 17;9(8):396. doi: 10.3390/bioengineering9080396 (PMC9405174; doi:10.3390/bioengineering9080396)
Supplement: Supplementary file 1 [file bioengineering-09-00396-s001.zip › bioengineering-1806841-supplementary.pdf]

**Table S1.** The hub genes in Turquoise module and Light cyan module and 67 hub genes related to lung metastasis coming from unit of Turquoise module and Light cyan module.

| Turquoise module hub genes | Light cyan module hub genes | 67 hub genes |
|---|---|---|
| AP2S1 | ARF5 | ARF5 |
| ARPC4 | ATP5MF | ATP5MF |
| ATP5MC2 | ATP6V1F | FASTK |
| ATP6V1F | FASTK | PDAP1 |
| BCL7C | PDAP1 | POLR2J |
| BRK1 | POLR2J | SSBP1 |
| CCDC12 | PTGES2 | AP2S1 |
| CD2BP2 | SSBP1 | ARPC4 |
| CIB1 | | ATP5MC2 |
| COMMD4 | | ATP6V1F |
| CSNK2B | | BCL7C |
| DYNLRB1 | | BRK1 |
| EXOSC5 | | CCDC12 |
| FBL | | CD2BP2 |
| FKBP1A | | CIB1 |
| HCFC1R1 | | COMMD4 |
| HRAS | | CSNK2B |
| IMP4 | | DYNLRB1 |
| MRPL14 | | EXOSC5 |
| MRPL17 | | FBL |
| MRPS26 | | FKBP1A |
| MZT2A | | HCFC1R1 |
| MZT2B | | HRAS |
| NDUFB11 | | IMP4 |
| NEDD8 | | MRPL14 |
| NME1 | | MRPL17 |
| NOP10 | | MRPS26 |
| NOSIP | | MZT2A |
| NSUN5 | | MZT2B |
| NUTF2 | | NDUFB11 |
| PFDN5 | | NEDD8 |
| PLPP3 | | NME1 |
| PMF1 | | NOP10 |
| POLR2H | | NOSIP |
| POLR2L | | NSUN5 |
| PSENEN | | NUTF2 |
| PTGES2 | | PFDN5 |
| PTRH2 | | PLPP3 |
| RALY | | PMF1 |
| RPL14 | | POLR2H |
| RPL18 | | POLR2L |
| RPL24 | | PSENEN |
| RPL27A | | PTGES2 |
| RPL32 | | PTRH2 |
| RPL35A | | RALY |
| RPL37 | | RPL14 |
| RPL38 | | RPL18 |
| RPLP1 | | RPL24 |

| | |
|---|---|
| RPLP2 | RPL27A |
| RPS11 | RPL32 |
| RPS15 | RPL35A |
| RPS16 | RPL37 |
| RUVBL2 | RPL38 |
| SERF2 | RPLP1 |
| SNRPA | RPLP2 |
| SNU13 | RPS11 |
| SSNA1 | RPS15 |
| TAF10 | RPS16 |
| TIMM17B | RUVBL2 |
| TMEM101 | SERF2 |
| TRIR | SNRPA |
| | SNU13 |
| | SSNA1 |
| | TAF10 |
| | TIMM17B |
| | TMEM101 |
| | TRIR |

**Table S2.** The expression of 67 genes between normal liver tissues and hepatocellular carcinoma tissues.

| Genes | Normal Number | Tumor Number | Normal Mean | Tumor Mean | *P* |
|---|---|---|---|---|---|
| AP2S1 | 47 | 370 | 4.236174053 | 4.743848669 | 1.63E-16 |
| ARF5 | 47 | 370 | 4.327995933 | 4.883097398 | 2.92E-18 |
| ARPC4 | 47 | 370 | 3.512863813 | 4.078881997 | 1.23E-17 |
| ATP5MC2 | 47 | 370 | 4.262816387 | 4.930182324 | 1.60E-22 |
| ATP5MF | 47 | 370 | 3.979963092 | 4.843541589 | 5.63E-26 |
| ATP6V1F | 47 | 370 | 4.293143721 | 5.20576096 | 2.62E-24 |
| BCL7C | 47 | 370 | 2.118654266 | 2.81385153 | 5.00E-19 |
| BRK1 | 47 | 370 | 4.791267292 | 5.283072575 | 6.96E-18 |
| CCDC12 | 47 | 370 | 2.349026338 | 2.886978 | 1.57E-17 |
| CD2BP2 | 47 | 370 | 2.673408624 | 3.475962989 | 9.08E-22 |
| CIB1 | 47 | 370 | 4.902221447 | 5.286295675 | 6.40E-11 |
| COMMD4 | 47 | 370 | 2.459161885 | 3.378739607 | 4.40E-26 |
| CSNK2B | 47 | 370 | 3.228806047 | 4.018508935 | 2.23E-22 |
| DYNLRB1 | 47 | 370 | 3.557521366 | 4.439422499 | 2.68E-25 |
| EXOSC5 | 47 | 370 | 2.831487938 | 3.685862191 | 8.82E-23 |
| FASTK | 47 | 370 | 3.177448077 | 4.080366239 | 2.47E-25 |
| FBL | 47 | 370 | 3.766325498 | 4.686045005 | 5.37E-22 |
| FKBP1A | 47 | 370 | 3.993354956 | 4.779695157 | 1.75E-22 |
| HCFC1R1 | 47 | 370 | 3.09895519 | 3.848124907 | 1.22E-20 |
| HRAS | 47 | 370 | 2.462832492 | 3.435088809 | 6.42E-25 |
| IMP4 | 47 | 370 | 3.162838782 | 3.641644946 | 7.75E-19 |
| MRPL14 | 47 | 370 | 4.55426441 | 5.202250606 | 2.49E-18 |
| MRPL17 | 47 | 370 | 2.907411014 | 3.614999108 | 5.51E-22 |
| MRPS26 | 47 | 370 | 4.232530675 | 4.773164023 | 1.02E-16 |
| MZT2A | 47 | 370 | 2.303094184 | 2.946016908 | 4.84E-14 |
| MZT2B | 47 | 370 | 3.88218848 | 4.412230407 | 1.94E-12 |
| NDUFB11 | 47 | 370 | 4.780598304 | 5.245927222 | 1.47E-13 |
| NEDD8 | 47 | 370 | 2.975431022 | 3.658874492 | 6.68E-25 |
| NME1 | 47 | 370 | 2.566431934 | 3.668551938 | 6.41E-24 |

| | | | | | |
|---|---|---|---|---|---|
| NOP10 | 47 | 370 | 4.974046135 | 5.556391615 | 4.43E-20 |
| NOSIP | 47 | 370 | 2.597752906 | 3.241833468 | 1.81E-19 |
| NSUN5 | 47 | 370 | 2.008022182 | 2.987134009 | 4.53E-25 |
| NUTF2 | 47 | 370 | 2.95722181 | 3.686968526 | 1.14E-23 |
| PDAP1 | 47 | 370 | 3.629840729 | 4.352080924 | 1.21E-25 |
| PFDN5 | 47 | 370 | 4.32326544 | 4.858788929 | 1.96E-17 |
| PLPP3 | 47 | 370 | 4.401122088 | 3.636643709 | 1.03E-13 |
| PMF1 | 47 | 370 | 2.754207444 | 3.616879903 | 5.24E-22 |
| POLR2H | 47 | 370 | 2.701028445 | 3.428003316 | 2.34E-22 |
| POLR2J | 47 | 370 | 3.656717386 | 4.335845913 | 2.30E-21 |
| POLR2L | 47 | 370 | 4.683328546 | 5.390286645 | 1.64E-20 |
| PSENEN | 47 | 370 | 3.27931317 | 3.883106072 | 1.08E-18 |
| PTGES2 | 47 | 370 | 2.904104984 | 3.682680475 | 3.55E-22 |
| PTRH2 | 47 | 370 | 1.960294131 | 2.66144024 | 1.69E-21 |
| RALY | 47 | 370 | 2.682623331 | 3.588081996 | 1.65E-25 |
| RPL14 | 47 | 370 | 4.087396049 | 4.802851689 | 3.03E-19 |
| RPL18 | 47 | 370 | 4.98737278 | 5.66695777 | 7.31E-17 |
| RPL24 | 47 | 370 | 5.31528629 | 5.888534047 | 4.96E-15 |
| RPL27A | 47 | 370 | 4.574013722 | 5.206954748 | 5.54E-16 |
| RPL32 | 47 | 370 | 5.301494139 | 5.946566872 | 8.16E-15 |
| RPL35A | 47 | 370 | 4.76989948 | 5.507470459 | 1.75E-19 |
| RPL37 | 47 | 370 | 4.381715346 | 5.144606041 | 4.43E-20 |
| RPL38 | 47 | 370 | 4.411329953 | 5.312217286 | 3.69E-22 |
| RPLP1 | 47 | 370 | 5.774536234 | 6.615128266 | 6.65E-22 |
| RPLP2 | 47 | 370 | 5.647896973 | 6.458490313 | 1.81E-19 |
| RPS11 | 47 | 370 | 6.536125455 | 7.208685787 | 2.70E-18 |
| RPS15 | 47 | 370 | 4.779601931 | 5.460213091 | 5.95E-17 |
| RPS16 | 47 | 370 | 5.543713865 | 6.321576168 | 4.17E-20 |
| RUVBL2 | 47 | 370 | 3.191600964 | 3.966654395 | 4.32E-24 |
| SERF2 | 47 | 370 | 4.480006043 | 5.105189806 | 3.79E-20 |
| SNRPA | 47 | 370 | 2.889018825 | 3.823946285 | 4.65E-26 |
| SNU13 | 47 | 370 | 3.670046745 | 4.348472223 | 2.17E-22 |
| SSBP1 | 47 | 370 | 2.666161325 | 3.30996936 | 8.43E-24 |
| SSNA1 | 47 | 370 | 3.749301352 | 4.537302589 | 5.11E-22 |
| TAF10 | 47 | 370 | 2.427629707 | 3.233120617 | 3.26E-26 |
| TIMM17B | 47 | 370 | 2.946945636 | 3.712441939 | 9.73E-24 |
| TMEM101 | 47 | 370 | 2.43906996 | 3.408766207 | 6.86E-25 |
| TRIR | 47 | 370 | 4.960609885 | 5.204161076 | 7.26E-06 |

normal: normal liver tissues. tumor: hepatocellular carcinoma tissues. Statistical analysis: Wilcoxon rank sum test. Mean: log (TPM+1).

**Table S3.** The expression of 67 genes between hepatocellular carcinoma tissues without metastasis and hepatocellular carcinoma tissues with lung metastasis.

| Genes | Non Number | Met Number | Non Mean | Met Mean | *P* |
|---|---|---|---|---|---|
| ARF5 | 13 | 18 | 4.570005153 | 5.042063047 | 0.000353 |
| ARPC4 | 13 | 18 | 3.867000476 | 4.187036753 | 0.0138 |
| ATP5MC2 | 13 | 18 | 4.720339159 | 5.123867245 | 0.00365 |
| ATP5MF | 13 | 18 | 4.611469347 | 4.995937259 | 0.0247 |
| ATP6V1F | 13 | 18 | 4.868319527 | 5.414773443 | 0.00233 |
| AP2S1 | 13 | 18 | 4.541733219 | 4.893841487 | 0.0564 |
| BCL7C | 13 | 18 | 2.507078455 | 3.072101564 | 0.00315 |
| BRK1 | 13 | 18 | 5.105341375 | 5.371575494 | 0.0175 |
| CCDC12 | 13 | 18 | 2.643539156 | 3.020931865 | 0.0221 |

| | | | | | |
|---|---|---|---|---|---|
| CD2BP2 | 13 | 18 | 3.229942997 | 3.60019467 | 0.0197 |
| CIB1 | 13 | 18 | 5.014628908 | 5.586124327 | 6.66E-05 |
| COMMD4 | 13 | 18 | 3.185341715 | 3.597329975 | 0.0156 |
| CSNK2B | 13 | 18 | 3.784803156 | 4.327940775 | 0.0156 |
| DYNLRB1 | 13 | 18 | 4.200194269 | 4.652276939 | 0.0247 |
| EXOSC5 | 13 | 18 | 3.367956056 | 3.895556975 | 0.0156 |
| FASTK | 13 | 18 | 3.748866306 | 4.285815525 | 6.66E-05 |
| FBL | 13 | 18 | 4.318137339 | 4.830185251 | 0.0138 |
| FKBP1A | 13 | 18 | 4.550502823 | 4.848077468 | 0.0512 |
| HCFC1R1 | 13 | 18 | 3.63991686 | 4.119581873 | 0.0122 |
| HRAS | 13 | 18 | 3.156390634 | 3.61431002 | 0.0512 |
| IMP4 | 13 | 18 | 3.45862231 | 3.781152885 | 0.00951 |
| MRPL14 | 13 | 18 | 5.024049492 | 5.541494188 | 0.0197 |
| MRPL17 | 13 | 18 | 3.325408211 | 3.912309672 | 0.00365 |
| MRPS26 | 13 | 18 | 4.509144071 | 4.850307112 | 0.0197 |
| MZT2A | 13 | 18 | 2.662547912 | 3.188033805 | 0.0122 |
| MZT2B | 13 | 18 | 4.063435369 | 4.654737502 | 0.00123 |
| NDUFB11 | 13 | 18 | 5.023454614 | 5.458779467 | 0.0138 |
| NEDD8 | 13 | 18 | 3.450410073 | 3.762364605 | 0.0122 |
| NME1 | 13 | 18 | 3.332932294 | 3.910559124 | 0.0175 |
| NOP10 | 13 | 18 | 5.385986629 | 5.801504318 | 0.0156 |
| NOSIP | 13 | 18 | 3.045587338 | 3.405222231 | 0.062 |
| NSUN5 | 13 | 18 | 2.77759885 | 3.170719105 | 0.00233 |
| NUTF2 | 13 | 18 | 3.493825926 | 3.812083437 | 0.0156 |
| PDAP1 | 13 | 18 | 4.148651301 | 4.407911099 | 0.0138 |
| PFDN5 | 13 | 18 | 4.650344099 | 4.980768055 | 0.0156 |
| PLPP3 | 13 | 18 | 3.918602329 | 3.22101653 | 0.0122 |
| PMF1 | 13 | 18 | 3.340291981 | 3.941336975 | 0.0138 |
| POLR2H | 13 | 18 | 3.25260968 | 3.650255739 | 0.0197 |
| POLR2J | 13 | 18 | 4.044241308 | 4.478988521 | 0.0122 |
| POLR2L | 13 | 18 | 5.148515762 | 5.552215515 | 0.00951 |
| PSENEN | 13 | 18 | 3.593537592 | 4.08091174 | 0.0017 |
| PTGES2 | 13 | 18 | 3.376898098 | 3.818746055 | 0.0138 |
| PTRH2 | 13 | 18 | 2.473991838 | 2.94878903 | 0.0175 |
| RALY | 13 | 18 | 3.391126471 | 3.754746032 | 0.0307 |
| RPL14 | 13 | 18 | 4.533568365 | 4.96338156 | 0.00835 |
| RPL18 | 13 | 18 | 5.33352164 | 5.866520285 | 0.00271 |
| RPL24 | 13 | 18 | 5.612584833 | 6.03869918 | 0.00365 |
| RPL27A | 13 | 18 | 4.985306181 | 5.482903655 | 0.00835 |
| RPL32 | 13 | 18 | 5.662555965 | 6.104706371 | 0.00732 |
| RPL35A | 13 | 18 | 5.2403584 | 5.659651209 | 0.0138 |
| RPL37 | 13 | 18 | 4.833223546 | 5.311903917 | 0.0017 |
| RPL38 | 13 | 18 | 4.990707614 | 5.518120271 | 0.00835 |
| RPLP1 | 13 | 18 | 6.338282882 | 6.858418654 | 0.00315 |
| RPLP2 | 13 | 18 | 6.166598336 | 6.704891944 | 0.00835 |
| RPS11 | 13 | 18 | 6.9565856 | 7.412949111 | 0.0108 |
| RPS15 | 13 | 18 | 5.167375937 | 5.652052976 | 0.00732 |
| RPS16 | 13 | 18 | 6.020542409 | 6.402853846 | 0.0138 |
| RUVBL2 | 13 | 18 | 3.726873226 | 4.096748552 | 0.0197 |
| SERF2 | 13 | 18 | 4.88596157 | 5.38366832 | 0.00365 |
| SNRPA | 13 | 18 | 3.523485441 | 3.950776983 | 0.0247 |
| SNU13 | 13 | 18 | 4.15547317 | 4.523124207 | 0.0276 |
| SSBP1 | 13 | 18 | 3.108569266 | 3.386831786 | 0.0342 |

| | | | | | |
|---|---|---|---|---|---|
| SSNA1 | 13 | 18 | 4.210373035 | 4.636069975 | 0.0138 |
| TAF10 | 13 | 18 | 3.056271973 | 3.479408001 | 0.00271 |
| TIMM17B | 13 | 18 | 3.476594046 | 3.978728866 | 0.00315 |
| TMEM101 | 13 | 18 | 3.068256891 | 3.539063345 | 0.0221 |
| TRIR | 13 | 18 | 4.938279043 | 5.345218226 | 0.00199 |

non: hepatocellular carcinoma tissues without metastasis. met: hepatocellular carcinoma tissues with lung metastasis. Statistical analysis: Wilcoxon rank sum test. Mean: log (TPM+1).

**Table S4.** 53 genes correlated to FBL with absolute coefficient > 0.4 in TCGA, GSE14520 and PDC000198.

| gene1 | gene2 | TCGA | | | GSE14520 | | | PDC000198 | | |
|---|---|---|---|---|---|---|---|---|---|---|
| | | cor_r | *P* value | FDR | cor_r | pvalue | FDR | cor_r | *P* value | FDR |
| FBL | ACADL | −0.565 | 1.52E−36 | 1.40E−35 | −0.521 | 1.30E−18 | 2.30E−16 | −0.426 | 1.13E−08 | 2.41E−07 |
| FBL | ALDH9A1 | −0.420 | 2.98E−19 | 1.07E−18 | −0.464 | 1.34E−14 | 9.88E−13 | −0.463 | 3.74E−10 | 1.20E−08 |
| FBL | ALDOB | −0.481 | 1.48E−25 | 7.57E−25 | −0.433 | 1.02E−12 | 4.90E−11 | −0.554 | 1.17E−14 | 9.35E−13 |
| FBL | APEX1 | 0.601 | 2.61E−42 | 3.41E−41 | 0.533 | 1.63E−19 | 3.43E−17 | 0.403 | 8.15E−08 | 1.36E−06 |
| FBL | ATIC | 0.719 | 1.38E−67 | 7.66E−66 | 0.533 | 1.44E−19 | 3.13E−17 | 0.444 | 2.27E−09 | 5.72E−08 |
| FBL | BYSL | 0.621 | 6.60E−46 | 1.07E−44 | 0.423 | 3.81E−12 | 1.56E−10 | 0.563 | 3.44E−15 | 3.05E−13 |
| FBL | BZW2 | 0.585 | 1.42E−39 | 1.57E−38 | 0.546 | 1.49E−20 | 3.55E−18 | 0.528 | 3.33E−13 | 2.04E−11 |
| FBL | CPSF6 | 0.502 | 5.40E−28 | 3.16E−27 | 0.479 | 1.48E−15 | 1.44E−13 | 0.500 | 7.69E−12 | 3.50E−10 |
| FBL | DAO | −0.465 | 8.52E−24 | 3.93E−23 | −0.500 | 4.72E−17 | 6.23E−15 | −0.453 | 9.81E−10 | 2.76E−08 |
| FBL | DDX50 | 0.517 | 7.62E−30 | 4.94E−29 | 0.463 | 1.55E−14 | 1.12E−12 | 0.482 | 5.40E−11 | 2.13E−09 |
| FBL | EIF3D | 0.744 | 1.38E−74 | 1.12E−72 | 0.609 | 1.68E−26 | 7.46E−24 | 0.449 | 1.43E−09 | 3.83E−08 |
| FBL | EIF3E | 0.562 | 5.13E−36 | 4.58E−35 | 0.472 | 4.41E−15 | 3.82E−13 | 0.483 | 5.23E−11 | 2.07E−09 |
| FBL | EIF3H | 0.600 | 4.40E−42 | 5.67E−41 | 0.449 | 1.15E−13 | 6.82E−12 | 0.432 | 7.03E−09 | 1.59E−07 |
| FBL | EIF6 | 0.743 | 2.73E−74 | 2.16E−72 | 0.449 | 1.19E−13 | 6.98E−12 | 0.411 | 4.10E−08 | 7.62E−07 |
| FBL | ETFDH | −0.678 | 2.42E−57 | 7.24E−56 | −0.410 | 1.91E−11 | 6.63E−10 | −0.452 | 1.10E−09 | 3.01E−08 |
| FBL | ETNPPL | −0.587 | 5.81E−40 | 6.63E−39 | −0.452 | 7.20E−14 | 4.51E−12 | −0.417 | 2.51E−08 | 4.94E−07 |
| FBL | GPD1 | −0.572 | 1.14E−37 | 1.13E−36 | −0.470 | 5.87E−15 | 4.92E−13 | −0.497 | 1.06E−11 | 4.81E−10 |
| FBL | GYS2 | −0.633 | 5.54E−48 | 1.02E−46 | −0.501 | 4.20E−17 | 5.61E−15 | −0.417 | 2.52E−08 | 4.96E−07 |
| FBL | HAGH | −0.412 | 1.73E−18 | 5.91E−18 | −0.466 | 9.53E−15 | 7.53E−13 | −0.439 | 3.78E−09 | 9.04E−08 |
| FBL | HAO2 | −0.515 | 1.44E−29 | 9.22E−29 | −0.480 | 1.28E−15 | 1.27E−13 | −0.518 | 1.09E−12 | 5.98E−11 |
| FBL | HMGA1 | 0.656 | 1.43E−52 | 3.39E−51 | 0.470 | 5.57E−15 | 4.70E−13 | 0.462 | 4.15E−10 | 1.30E−08 |
| FBL | HNRNPA3 | 0.552 | 1.15E−34 | 9.71E−34 | 0.475 | 2.82E−15 | 2.59E−13 | 0.496 | 1.31E−11 | 5.87E−10 |
| FBL | HNRNPH3 | 0.420 | 2.76E−19 | 9.95E−19 | 0.407 | 3.00E−11 | 1.01E−09 | 0.409 | 5.02E−08 | 9.00E−07 |
| FBL | HNRNPM | 0.597 | 1.43E−41 | 1.79E−40 | 0.576 | 2.83E−23 | 8.99E−21 | 0.477 | 9.57E−11 | 3.56E−09 |
| FBL | HSD17B6 | −0.522 | 1.41E−30 | 9.53E−30 | −0.446 | 1.81E−13 | 1.04E−11 | −0.412 | 3.69E−08 | 6.96E−07 |
| FBL | IGF2BP2 | 0.522 | 1.51E−30 | 1.01E−29 | 0.440 | 3.92E−13 | 2.06E−11 | 0.450 | 1.32E−09 | 3.56E−08 |
| FBL | ILF3 | 0.608 | 1.36E−43 | 1.90E−42 | 0.429 | 1.74E−12 | 7.77E−11 | 0.463 | 3.81E−10 | 1.21E−08 |
| FBL | IMPDH2 | 0.809 | 8.39E−98 | 1.80E−95 | 0.732 | 1.05E−42 | 2.17E−39 | 0.511 | 2.41E−12 | 1.24E−10 |
| FBL | IPO4 | 0.734 | 1.27E−71 | 8.79E−70 | 0.456 | 4.45E−14 | 2.93E−12 | 0.442 | 2.79E−09 | 6.87E−08 |
| FBL | LRRC1 | 0.545 | 1.28E−33 | 1.01E−32 | 0.433 | 1.05E−12 | 5.01E−11 | 0.420 | 2.03E−08 | 4.07E−07 |
| FBL | NAT10 | 0.594 | 3.90E−41 | 4.75E−40 | 0.426 | 2.54E−12 | 1.08E−10 | 0.575 | 6.96E−16 | 6.77E−14 |
| FBL | NCL | 0.567 | 9.00E−37 | 8.36E−36 | 0.521 | 1.31E−18 | 2.30E−16 | 0.708 | 1.90E−26 | 9.00E−24 |
| FBL | NLE1 | 0.737 | 1.09E−72 | 7.91E−71 | 0.497 | 8.56E−17 | 1.05E−14 | 0.459 | 5.89E−10 | 1.79E−08 |
| FBL | NPM1 | 0.636 | 1.26E−48 | 2.42E−47 | 0.500 | 5.30E−17 | 6.78E−15 | 0.512 | 1.99E−12 | 1.06E−10 |
| FBL | NSA2 | 0.562 | 4.85E−36 | 4.34E−35 | 0.501 | 4.10E−17 | 5.53E−15 | 0.476 | 1.07E−10 | 3.89E−09 |
| FBL | PDCD11 | 0.595 | 2.94E−41 | 3.61E−40 | 0.510 | 9.90E−18 | 1.46E−15 | 0.730 | 8.59E−29 | 8.71E−26 |
| FBL | PES1 | 0.763 | 1.24E−80 | 1.34E−78 | 0.495 | 1.13E−16 | 1.36E−14 | 0.632 | 8.17E−20 | 1.18E−17 |
| FBL | PFKFB1 | −0.485 | 5.60E−26 | 2.93E−25 | −0.471 | 5.02E−15 | 4.29E−13 | −0.429 | 8.98E−09 | 2.00E−07 |
| FBL | POLR1D | 0.558 | 1.58E−35 | 1.38E−34 | 0.434 | 9.65E−13 | 4.65E−11 | 0.410 | 4.67E−08 | 8.46E−07 |
| FBL | PRMT1 | 0.752 | 3.47E−77 | 3.15E−75 | 0.500 | 5.14E−17 | 6.71E−15 | 0.404 | 7.57E−08 | 1.29E−06 |
| FBL | RBM12 | 0.407 | 4.26E−18 | 1.43E−17 | 0.578 | 2.13E−23 | 6.94E−21 | 0.564 | 3.02E−15 | 2.71E−13 |

| | | | | | | | | | | |
|---|---|---|---|---|---|---|---|---|---|---|
| FBL | RPP30 | 0.715 | 1.75E−66 | 9.03E−65 | 0.444 | 2.34E−13 | 1.29E−11 | 0.414 | 3.29E−08 | 6.27E−07 |
| FBL | RSL1D1 | 0.556 | 3.38E−35 | 2.93E−34 | 0.449 | 1.15E−13 | 6.80E−12 | 0.643 | 1.20E−20 | 1.99E−18 |
| FBL | RUVBL2 | 0.799 | 1.18E−93 | 2.11E−91 | 0.576 | 3.30E−23 | 9.99E−21 | 0.555 | 1.07E−14 | 8.81E−13 |
| FBL | SLC10A1 | −0.570 | 2.29E−37 | 2.22E−36 | −0.502 | 3.65E−17 | 5.03E−15 | −0.427 | 1.10E−08 | 2.37E−07 |
| FBL | SLC46A3 | −0.606 | 3.15E−43 | 4.30E−42 | −0.613 | 7.37E−27 | 3.66E−24 | −0.521 | 7.69E−13 | 4.30E−11 |
| FBL | SSB | 0.550 | 2.84E−34 | 2.33E−33 | 0.512 | 6.41E−18 | 9.70E−16 | 0.550 | 1.87E−14 | 1.39E−12 |
| FBL | TAT | −0.602 | 1.95E−42 | 2.57E−41 | −0.453 | 6.46E−14 | 4.17E−12 | −0.418 | 2.42E−08 | 4.79E−07 |
| FBL | TPD52L2 | 0.694 | 3.64E−61 | 1.38E−59 | 0.441 | 3.78E−13 | 1.99E−11 | 0.454 | 9.06E−10 | 2.59E−08 |
| FBL | TRIM28 | 0.805 | 3.29E−96 | 6.58E−94 | 0.597 | 3.22E−25 | 1.29E−22 | 0.443 | 2.61E−09 | 6.46E−08 |
| FBL | TRMT1 | 0.735 | 6.75E−72 | 4.71E−70 | 0.451 | 9.00E−14 | 5.44E−12 | 0.471 | 1.69E−10 | 5.96E−09 |
| FBL | WDR46 | 0.744 | 9.19E−75 | 7.48E−73 | 0.435 | 7.89E−13 | 3.87E−11 | 0.720 | 1.07E−27 | 7.62E−25 |
| FBL | XRCC6 | 0.683 | 1.17E−58 | 3.75E−57 | 0.415 | 1.05E−11 | 3.95E−10 | 0.554 | 1.14E−14 | 9.31E−13 |
